# The Impact of Postponed Fertility Treatment on the Sexual Health of Infertile Patients Owing to the COVID-19 Pandemic

**DOI:** 10.3389/fmed.2021.730994

**Published:** 2021-12-10

**Authors:** Meng Dong, Shanshan Wu, Yanqiang Tao, Feifei Zhou, Jichun Tan

**Affiliations:** ^1^Center of Reproductive Medicine, Shengjing Hospital of China Medical University, Shenyang, China; ^2^Key Laboratory of Reproductive Dysfunction Diseases and Fertility Remodeling of Liaoning, Shenyang, China; ^3^School of Life Sciences, China Medical University, Shenyang, China; ^4^Beijing Key Laboratory of Applied Experimental Psychology, Faculty of Psychology, Beijing Normal University, Beijing, China

**Keywords:** COVID-19, infertility, postponed fertility treatment, sexual health, psychological health, SEM

## Abstract

**Background:** With the onset of the coronavirus disease 2019 (COVID-19) pandemic at the beginning of 2020, all non-essential medical treatments were suspended, including fertility treatments. As a unique group in society, patients with infertility may be more sensitive and vulnerable in the face of pressure and crisis. However, to the best of our knowledge, there have been no reports on the influence of postponed fertility treatment on the sexual health of infertile patients owing to COVID-19. Therefore, this study aimed to investigate whether postponed fertility treatment resulting from COVID-19 affects the sexual health of patients with infertility.

**Methods:** A total of 1,442 participants were included for analysis in this large-scale study. Those with postponed fertility treatment were categorised as group A (*n* = 474), whereas those whose fertility treatment was not delayed were in group B (*n* = 968). The sexual health and psychological well-being were compared between the two groups.

**Results:** The total Female Sexual Function Index score and five domains of female sexual function (arousal ability, vaginal lubrication, orgasm, satisfaction, and coital pain) were significantly lower in group A than those in group B (*p* < 0.05). The International Index of Erectile Dysfunction score and Premature Ejaculation Diagnostic Tool score were significantly higher in group A than those in group B (*p* < 0.05).

**Conclusions:** Delaying fertility treatment obviously affects patients' sexual and mental health. Through a structural equation model, we observed that postponed fertility treatment mediates sexual health by regulating psychological distress and couple relationship quality.

## Introduction

Currently, COVID-19 has been one of the most serious infectious diseases in history. It has brought unprecedented challenges to the world given its high infectivity rate, high mortality rate, and an uncertain timeline for its complete resolution (1, 2). In the context of a global pandemic, individuals in a society may experience deep emotional traumas (3), such as anxiety, depression, posttraumatic stress disorder, and negative societal behaviours (4), all of which could result in stress-related illnesses (5). Studies have shown that COVID-19 has had a serious impact on the general population's mental, psychological, and sexual health, as well as interpersonal relationships (3, 4, 6–8).

Infertility is a serious condition that affects 8–12% of couples of reproductive age and harms the physical and mental health of those affected (9). With the onset of this global pandemic at the beginning of 2020, all non-essential medical treatments were suspended, including fertility treatments. In the early stages of COVID-19, the American Society for Reproductive Medicine (ASRM) and the European Society of Human Reproduction and Embryology (ESHRE) required that reproductive health care should be stopped except in the most urgent of cases (9). As a unique group in society, individuals with infertility may be more sensitive and vulnerable in the face of pressure and crisis (10). Recently, published data indicated that interruption of fertility treatment may increase psychological distress (i.e., anxiety or depression) and cause individuals to feel helpless and hopeless because of the unfulfilled desire for fertility and uncertainty about the future (10–13).

Their sexual health may also be affected by this psychosocial distress (14). Although some studies have explored the impact of the COVID-19 pandemic on the mental health of patients with infertility (10–13), research still needs to be conducted on the impact on sexual health, which is a vital aspect of daily well-being (15–17). The sexual health of patients with infertility is a topic of great concern; it is also an important aspect for the treatment of infertility. Many studies found that infertility is a risk factor for sexual dysfunction and the incidence of sexual dysfunction in patients with infertility is significantly higher than that of the fertile population (18, 19). Further, studies confirmed that during the COVID-19 pandemic, people's sexual behaviour has also changed resulting from changes in emotions (6, 7, 14). Recent investigations of the pandemic's impact on sexual health have mostly focused on the general population and have shown that the pandemic has reduced the quality of sexual life and caused a change in the frequency of sexual intercourse (6, 7, 15, 20). Based on the above studies, we hypothesise that the same would be the case for patients with infertility. However, no reports currently exist on the changes in sexual health of this population. Therefore, this study aimed to determine whether postponed fertility treatment resulting from the COVID-19 pandemic had an impact on the psychological and sexual health of patients with infertility.

## Materials and Methods

### Participants

This study was conducted at the Reproductive Medical Centre of Shengjing Hospital of China Medical University. A total of 1,767 patients voluntarily participated in the survey between 1 July 2020 and 12 March 2021. All participants were recruited from patients being treated with assisted reproductive technology at the Reproductive Medical Centre. Participants were divided into two groups according to whether or not they experienced any postponed fertility treatment resulting from the COVID-19 pandemic. Group A comprised individuals who were diagnosed with infertility before the COVID-19 outbreak and whose fertility treatment was interrupted by the lockdown. The inclusion criterion for group A was postponement of fertility treatment for at least 3 months. Group B comprised infertile patients whose fertility treatments were not delayed. Infertility is defined as the inability to conceive after 1 year of unprotected intercourse (21). Patients previously diagnosed with sexual dysfunction and those taking medication that may affect their sexual function or mental state were excluded (e.g., selective serotonin reuptake inhibitors, tricyclic antidepressants, and phosphodiesterase type 5 inhibitors) (22).

### Questionnaire

A survey of sexual health, psychological well-being, and couple relationship quality of the patients with infertility during the COVID-19 pandemic was performed. Questionnaires were anonymous and confidential, and specific members of the research team explained the questions to the participants to ensure proper understanding. The questionnaires were completed in a private room after a visit to the Reproductive Medical Centre, and completed questionnaires were placed in a box and collected altogether.

The questionnaire was composed of four parts. The first part included several demographic details: age, body mass index (BMI), economic level, infertility duration, drug use, education levels, and living habits (frequency of physical exercise and smoking and drinking status). The second part focused on the COVID-19-related impact on changes in sexual behaviour, anxiety symptoms, couple relationships, and income. The following survey items were included: “Have you postponed fertility treatment due to the COVID-19 pandemic?”, “Have you become anxious because of the COVID-19 pandemic?”, “Are there any changes in your romantic relationships, sexual desire, frequency of intercourse, sexual satisfaction, frequency of masturbation, pornography use, and financial income due to the COVID-19 lockdown?”

The third part focused on the psychological health (including anxiety and depression symptoms) and couple relationships of the participants during the COVID-19 lockdown. These were evaluated using the Generalised Anxiety Disorder scale (GAD-7) (23), the Patient Health Questionnaire (PHQ-9) (24), and the Quality of Marriage Index (QMI) (25). The cut-off score of the GAD-7 was ≥10, which determined the presence of anxiety (23), and a PHQ-9 cut-off score of ≥10 was used to determine the presence of depression (24). We assessed their couple relationships using the QMI, a 6-item scale used to assess relationship quality, the cut-off score for which was <34, which indicated a low-quality couple relationship (25).

The fourth part included a survey of the participants' sexual health during the COVID-19 pandemic. The questions were adapted from validated sexual function questionnaires and a question on dyspareunia was also included. Female sexual function was assessed by the Female Sexual Function Index (FSFI), which comprises 19 items and six domains of female sexual function (desire, arousal, lubrication, orgasm, satisfaction, and coital pain) and is answered based on the participant's sexual status in the four preceding weeks (26). A total FSFI score of 23.45 (Chinese cut-off) or lower indicates that the woman might have sexual dysfunction (27, 28). The Cronbach's alpha values were ≥0.82 (26). Male sexual function was assessed using the International Index of Erectile Dysfunction (IIEF-15) and Premature Ejaculation Diagnostic Tool (PEDT) based on their sexual status in the four preceding weeks. The IIEF-15 includes 15 items and five domains of male sexual function (erectile function, orgasmic function, sexual desire, intercourse satisfaction, and overall satisfaction) with Cronbach's alpha values ≥0.91 (29, 30). The presence and severity of erectile dysfunction (ED) was based on the IIEF-EF domain score: 26–30 (no ED), 22–25 (mild ED), 17–21 (mild to moderate ED), 11–16 (moderate ED), and <11 (severe ED) (31). A PEDT score of ≤8 indicated no premature ejaculation (PE), scores of 9 and 10 indicated probable PE, and scores ≥11 indicated PE. Cronbach's alpha value was 0.78 (32).

### Statistical Analysis

Data analysis was performed using the SPSS statistical software (version 22.0; SPSS Inc., Chicago, IL, USA). Categorical variables were summarised with counts and percentages. Continuous variables were summarised with means and standard deviations (SDs). The Chi-square test was used to compare categorical data, and the independent *t*-test was used to compare numerical data. A two-tailed *p*-value of <0.05 indicated statistical significance.

Logistic regression was employed to explore the factors that affect sexual function (normal = 1, dysfunction = 0), the *p*-value, odds ratio (OR), and 95% confidence interval (CI) were evaluated. We performed a structural equation model (SEM) to assess the impact of postponed fertility treatment on study variables and drew a path diagram for each gender. We used the package of “lavaan” in R for calculating the effect of mediation. The overall fitting model and goodness-of-fit were evaluated with the following indices: ratio of χ^2^ values and degrees of freedom values (χ^2^/df), root mean square error of approximation (RMSEA), standardised root mean square residual (SRMR), goodness-of-fit index (GFI), normed fit index (NFI), and comparative fit index (CFI) (33).

### Ethical Approval

This study was approved by the Institutional Review Board for Research on Human Subjects (2020PS009F).

## Results

### Participants' Demographic Characteristics

Of the 1,767 respondents in this study, 325 were excluded because of incomplete questionnaires, and the response rate was 81.6% (1,442/1,767). Those with delayed fertility treatments resulting from lockdown comprised group A (*n* = 474), whereas those whose fertility treatments were not delayed comprised group B (*n* = 968). Table 1 shows the participants' demographic characteristics. The average age was 34.03 years for group A and 33.57 years for group B (*p* > 0.05). The average infertility duration of group A was 4.54 years and 4.04 years for group B (*p* < 0.05). There were no significant differences in BMI, education level, income level, stress level, and living habits (i.e., smoking, drinking, and physical exercise frequency) between the two groups (*p* > 0.05; Table 1).

**Table 1:** Demographic characteristics of the subject population.

	**Total (*n* = 1442)**	**Group A (*n* = 474)**	**Group B (*n* = 968)**	**Statistics**	***P*** **-value**
	**mean ± SD/*n* (%)**	**mean ± SD/*n* (%)**	**mean ± SD/*n* (%)**	***t/χ^2^***	
Age(years)	33.72 ± 4.97	34.03 ± 5.09	33.57 ± 4.91	*t* = 1.65	0.10
BMI (kg/m^2^)	24.43 ± 3.82	24.36 ± 3.82	24.47 ± 3.83	*t* = −0.48	0.64
**Gender**				*χ^2^* = 0.49	0.49
Male	615 (42.6)	196 (41.4)	419 (43.3)		
Female	827 (57.4)	278 (58.6)	549 (56.7)		
Duration of infertility	4.20 ± 3.25	4.54 ± 3.55	4.04 ± 3.08	*t* = 2.80	0.01^*^
**Income**				*χ^2^* = 0.21	0.90
Low	655 (45.4)	215 (45.4)	440 (45.5)		
Middle	637 (44.2)	212 (44.7)	425 (43.9)		
High	150 (10.4)	47 (9.9)	103 (10.6)		
**Education levels**				*χ^2^* = 3.16	0.37
≤ High school	481 (33.4)	169 (35.7)	312 (32.2)		
Specialized college	345 (23.9)	101 (21.3)	244 (25.2)		
University	502 (34.8)	166 (35.0)	336 (34.7)		
≥Postgraduate	114 (7.9)	38 (8.0)	76 (7.9)		
**Stress levels**				*χ^2^* = 3.59	0.47
Very high	92 (6.4)	29 (6.1)	63 (6.5)		
High	294 (20.4)	91 (19.2)	203 (21.0)		
General	803 (55.7)	278 (58.6)	525 (54.2)		
Low	189 (13.1)	60 (12.7)	129 (13.3)		
None	64 (4.4)	16 (3.4)	48 (5.0)		
**Physical exercise frequency**				*χ^2^* = 4.85	0.18
None	469 (32.5)	163 (34.4)	306 (31.6)		
<1 time a week	436 (30.2)	129 (27.2)	307 (31.7)		
1 time a week	410 (28.4)	145 (30.6)	265 (27.4)		
≥2 times a week	127 (8.8)	37 (7.8)	90 (9.3)		
**Smoking status**				*χ^2^* = 0.26	0.61
Smoker	305 (21.2)	104 (21.9)	201 (20.8)		
Non-smoker	1137 (78.8)	370 (78.1)	767 (79.2)		
**Drinking alcohol**				*χ^2^* = 8.15	0.09
Almost everyday	18 (1.2)	3 (0.6)	15 (1.5)		
Often	69 (4.8)	17 (3.6)	52 (5.4)		
Sometimes	211 (14.6)	80 (16.9)	131 (13.5)		
Rarely	549 (38.1)	189 (39.9)	360 (37.2)		
Never	595 (41.3)	185 (39.0)	410 (42.4)		

*SD, standard deviation; BMI, body mass index*.

* *p < 0.05*.

### COVID-19-Related Impact on Changes in Sexuality, Psychological Distress, and Couple Relationships

In terms of sexuality, patients in group A reported a higher rate of decrease in sexual desire, frequency of intercourse, and sexual satisfaction compared with group B (*p* < 0.01). The changes in alcohol consumption before sexual activity, frequency of masturbation and use of pornography were significantly different between the two groups (*p* < 0.01). For COVID-19-related anxiety, the severe anxiety rate in group A was significantly higher than that of group B (*p* < 0.01). The ratio of deterioration in the couple relationship in group A was higher than that of group B (*p* < 0.01; Table 2).

**Table 2:** COVID-19-related impact on infertile patients.

	**Total (*n* = 1,442)**	**Group A (*n* = 474)**	**Group B (*n* = 968)**	**Statistics**	***P*** **-value**
	***n* (%)**	***n* (%)**	***n* (%)**	***χ^2^***	
**COVID-19-related anxiety**				79.86	<0.01
Severe	158 (11.0)	86 (18.1)	72 (7.4)		
Slight	354 (24.5)	156 (32.9)	198 (20.5)		
None	930 (64.5)	232 (48.9)	698 (72.1)		
**Partner relationship**				196.89	<0.01
Worse	115 (8.0)	105 (22.2)	10 (1.0)		
Unchanged	1006 (69.8)	266 (56.1)	740 (76.4)		
Better	321 (22.3)	103 (21.7)	218 (22.5)		
**Sexual desire**				99.10	<0.01
Decreased	187 (13.0)	118 (24.9)	69 (7.1)		
Unchanged	1185 (82.2)	324 (68.4)	861 (88.9)		
Increased	70 (4.9)	32 (6.8)	38 (3.9)		
**Sexual frequency**				101.18	<0.01
Decreased	222 (15.4)	135 (28.5)	87 (9.0)		
Unchanged	1137 (78.8)	304 (64.1)	833 (86.1)		
Increased	83 (5.8)	35 (7.4)	48 (5.0)		
**Sexual satisfaction**				153.97	<0.01
Decreased	172 (11.9)	127 (26.8)	45 (4.6)		
Unchanged	1212 (84.0)	323 (68.1)	889 (91.8)		
Increased	58 (4.0)	24 (5.1)	34 (3.5)		
**Drinking alcohol before sexual activity**				46.14	<0.01
Decreased	112 (7.8)	69 (14.6)	43 (4.4)		
Unchanged	1092 (75.7)	338 (71.3)	754 (77.9)		
Increased	238 (16.5)	67 (14.1)	171 (17.7)		
**Frequency of masturbation**				113.47	<0.01
Decreased	179 (12.4)	76 (16.0)	103 (10.6)		
Unchanged	610 (42.3)	170 (35.9)	440 (45.5)		
Increased	194 (13.5)	122 (25.7)	72 (7.4)		
None	459 (31.8)	106 (22.4)	353 (36.5)		
**Frequency of pornography use**				143.55	<0.01
Decreased	143 (9.9)	88 (18.6)	55 (5.7)		
Unchanged	467 (32.4)	108 (22.8)	359 (37.1)		
Increased	118 (8.2)	79 (16.7)	39 (4.0)		
None	714 (49.5)	199 (42.0)	515 (53.2)		
**Income changed**				58.25	<0.01
Decreased	742 (51.5)	309 (65.2)	433 (44.7)		
Unchanged	678 (47.0)	155 (32.7)	523 (54.0)		
Increased	22 (1.5)	10 (2.1)	12 (1.2)		

### Sexual Health During the COVID-19 Pandemic

For female sexual health, the total FSFI scores and scores under five domains of female sexual function (arousal ability, vaginal lubrication, orgasm, satisfaction, and coital pain) were significantly lower in group A than in group B (*p* < 0.05). Additionally, the incidence of sexual dysfunction in group A was significantly higher than in group B (*p* < 0.01). Moreover, the frequency of intercourse and incidence of dyspareunia was significantly different between the two groups (*p* < 0.05; Table 3).

**Table 3:** Female sexual health during COVID-19 pandemic.

	**Total (*n* = 827)**	**Group A (*n* = 278)**	**Group B (*n* = 549)**	**Statistics**	***P*** **-value**
	**Mean ± SD/*n* (%)**	**Mean ± SD/*n* (%)**	**Mean ± SD/*n* (%)**	***t/χ* ^2^**	
Age (years)	32.99 ± 4.63	33.22 ± 4.59	32.88 ± 4.64	*t* = 1.00	0.32
BMI (kg/m^2^)	23.42 ± 3.35	23.33 ± 3.23	23.47 ± 3.41	*t* = −0.54	0.59
Sexual activities frequency	4.65 ± 2.88	4.28 ± 2.99	4.84 ± 2.81	*t* = −2.61	0.01^*^
**FSFI total score**	26.53 ± 4.16	25.72 ± 4.72	26.93 ± 3.78	*t* = −3.99	<0.01
Sexual desire	3.36 ± 0.77	3.39 ± 0.83	3.34 ± 0.74	*t* = 0.95	0.34
Arousal ability	4.00 ± 0.97	3.89 ± 1.01	4.05 ± 0.95	*t* = −2.22	0.03^*^
Vaginal lubrication	5.11 ± 0.84	4.95 ± 0.93	5.19 ± 0.77	*t* = −3.93	<0.01
Orgasm	4.52 ± 1.01	4.34 ± 1.10	4.62 ± 0.95	*t* = −3.75	<0.01
Satisfaction	4.66 ± 0.95	4.56 ± 1.09	4.71 ± 0.87	*t* = −2.04	0.04^*^
Coital pain	4.89 ± 1.00	4.59 ± 1.18	5.04 ± 0.87	*t* = −6.19	<0.01
Incidence of sexual dysfunction	159 (19.2)	75 (27.0)	84 (15.3)	*χ^2^* = 16.21	<0.01
**Dyspareunia**				*χ^2^* = 11.17	<0.05
Almost always	5 (0.6)	2 (0.7)	3 (0.5)		
Usually	17 (2.1)	9 (3.2)	8 (1.5)		
Sometimes	138 (16.7)	57 (20.5)	81 (14.8)		
Rarely	189 (22.9)	57 (20.5)	132 (24.0)		
Very rarely	164 (19.8)	61 (21.9)	103 (18.8)		
Never	314 (38.0)	92 (33.1)	222 (40.4)		

*FSFI, Female Sexual Function Index*.

* *p < 0.05*.

For male sexual health, the total IIEF-15 scores, erectile function, orgasmic function, intercourse satisfaction, and overall satisfaction were significantly lower in group A than in group B (*p* < 0.05). The PEDT scores and PE incidence were significantly higher in group A than in group B (*p* < 0.01). The frequency of intercourse, incidence of ED, and dyspareunia were significantly different between the two groups (*p* < 0.05; Table 4).

**Table 4:** Male sexual health during COVID-19 pandemic.

	**Total (*n* = 615)**	**Group A (*n* = 196)**	**Group B (*n* = 419)**	**Statistics**	***P*** **-value**
	**Mean ± SD/*n* (%)**	**Mean ± SD/*n* (%)**	**Mean ± SD/*n* (%)**	***t/χ* ^2^**	
Age (years)	34.70 ± 5.25	35.17 ± 5.53	34.47 ± 5.11	*t* = 1.54	0.12
BMI (kg/m^2^)	25.79 ± 3.99	25.83 ± 4.11	25.77 ± 3.95	*t* = 0.15	0.88
Sexual activities frequency	5.15 ± 2.85	4.76 ± 2.92	5.33 ± 2.80	*t* = −2.35	0.02^*^
**IIEF-15 score**	56.27 ± 9.08	53.97 ± 10.78	57.35 ± 7.95	*t* = −4.37	<0.01
Erectile function	24.52 ± 4.23	23.62 ± 4.87	24.94 ± 3.83	*t* = −3.64	<0.01
Orgasmic function	7.72 ± 1.75	7.45 ± 1.88	7.84 ± 1.67	*t* = −2.60	0.01^*^
Sexual desire	6.32 ± 1.30	6.34 ± 1.42	6.32 ± 1.24	*t* = 0.24	0.81
Intercourse satisfaction	10.20 ± 2.37	9.82 ± 2.71	10.38 ± 2.17	*t* = −2.78	0.01^*^
Overall satisfaction	7.52 ± 1.90	6.74 ± 2.36	7.88 ± 1.52	*t* = −7.16	<0.01
**Incidence of ED**				*χ^2^* = 14.12	0.01^*^
No ED	316 (51.4)	85 (43.4)	231 (55.1)		
Mild ED	175 (28.5)	57 (29.1)	118 (28.2)		
Mild–moderate ED	87 (14.1)	34 (17.3)	53 (12.6)		
Moderate ED	35 (5.7)	19 (9.7)	16 (3.8)		
Severe ED	2 (0.3)	1 (0.5)	1 (0.2)		
PEDT score	5.46 ± 2.56	6.28 ± 2.99	5.08 ± 2.24	*t* = 5.54	<0.01
**Incidence of PE**				*χ^2^* = 41.44	<0.01
No PE	511 (83.1)	135 (68.9)	376 (89.7)		
Probable PE	71 (11.5)	41 (20.9)	30 (7.2)		
PE	33 (5.4)	20 (10.2)	13 (3.1)		
**Dyspareunia**				*χ^2^* = 15.40	0.01^*^
Almost always	8 (1.3)	3 (1.5)	5 (1.2)		
Usually	13 (2.1)	7 (3.6)	6 (1.4)		
Sometimes	114 (18.5)	51 (26.0)	63 (15.0)		
Rarely	116 (18.9)	35 (17.9)	81 (19.3)		
Very rarely	136 (22.1)	35 (17.9)	101 (24.1)		
Never	228 (37.1)	65 (33.2)	163 (38.9)		

*IIEF-15, International Index of Erectile Dysfunction; ED, Erectile dysfunction; PEDT, Premature Ejaculation Diagnostic Tool; PE, Premature ejaculation*.

* *p < 0.05*.

### Psychological Health and Quality of Marriage During the COVID-19 Pandemic

The GAD-7 scores and PHQ-9 scores were significantly higher in group A than in group B, regardless of gender (*p* < 0.01). Thus, the incidence of anxiety and depression were significantly higher in group A than in group B (*p* < 0.01). In addition, the QMI score was significantly lower in group A than in group B regardless of gender (*p* < 0.05; Table 5).

**Table 5:** Subject's psychological health and quality of couple relationship.

**Items**	**Female**				**Male**			
	**Group A**	**Group B**	**Statistics**	***P*1- value**	**Group A**	**Group B**	**Statistics**	***P*2*-* value**
	**(*n* = 278)**	**(*n* = 549)**			**(*n* = 196)**	**(*n* = 419)**		
GAD-7 score	8.36 ± 4.58	5.83 ± 1.96	*t* = 11.10	<0.01	8.06 ± 4.89	6.14 ± 2.70	*t* = 6.27	<0.01
Prevalence (%)	94 (33.8)	26 (4.7)	*χ^2^* = 125.78	<0.01	73 (37.2)	56 (13.4)	*χ^2^* = 45.94	<0.01
PHQ-9 score	10.55 ± 5.69	7.24 ± 2.48	*t* = 11.63	<0.01	10.40 ± 5.57	7.14 ± 3.02	*t* = 9.38	<0.01
Prevalence (%)	120 (43.2)	91 (16.6)	*χ^2^* = 68.66	<0.01	90 (45.9)	78 (18.6)	*χ^2^* = 50.14	<0.01
QMI- score	28.46 ± 7.82	29.50 ± 5.02	*t* = −2.32	0.02^*^	28.68 ± 8.92	33.83 ± 5.83	*t* = −8.55	<0.01

*GAD-7, Generalized Anxiety Disorder-7; PHQ-9, Patient Health Questionnaire-9; QMI, Quality of marriage index*.

* *P < 0.05*.

### Related Factors Affecting Sexual Dysfunction

The logistic regression analysis of the relevant factors that affect sexual dysfunction showed that male gender (OR 1.79, 95% CI: 1.23–2.61, *p* < 0.01), and depressive symptoms (OR 1.21, 95% CI: 1.08–1.35, *p* < 0.01) are risk factors for the occurrence of sexual dysfunction. High QMI score (OR 0.87, 95% CI: 0.84–0.89, *p* < 0.01) are protective factor for the occurrence of sexual dysfunction. However, postponed fertility treatment (OR 0.81, 95% CI: 0.57–1.14, *p* > 0.05) is not a risk factor for sexual dysfunction (Table 6).

**Table 6:** Logistics regression analysis of the relevant factors related to sexual dysfunction (normal = 1, dysfunction = 0).

**Items**	**OR**	**95% CI**	***P-*** **values**
**Postponed fertility treatment**			
No	1.00		
Yes	0.81	0.57–1.14	0.23
**Gender**			
Female	1.00		
Male	1.79	1.23–2.61	<0.01
Age	1.03	1.00–1.07	0.06
BMI	1.01	0.97–1.05	0.78
Infertility duration	0.97	0.93–1.02	0.23
Education	0.95	0.81–1.11	0.53
Frequency of sexual behaviours	0.99	0.93–1.06	0.77
Stress levels	0.99	0.83–1.18	0.93
Frequency of physical exercise	0.96	0.82–1.13	0.64
Income levels	1.16	0.91–1.47	0.22
**Smoking status**			
No	1.00		
Yes	0.85	0.56–1.29	0.44
Drinking status	1.08	0.90–1.29	0.43
Anxiety	0.91	0.80–1.04	0.18
Depression	1.21	1.08–1.35	<0.01
QMI	0.87	0.84–0.89	<0.01

### Relationship Between Postponed Fertility Treatments, Psychological Distress, Relationship Quality, and Sexual Health

**Figure 1:**
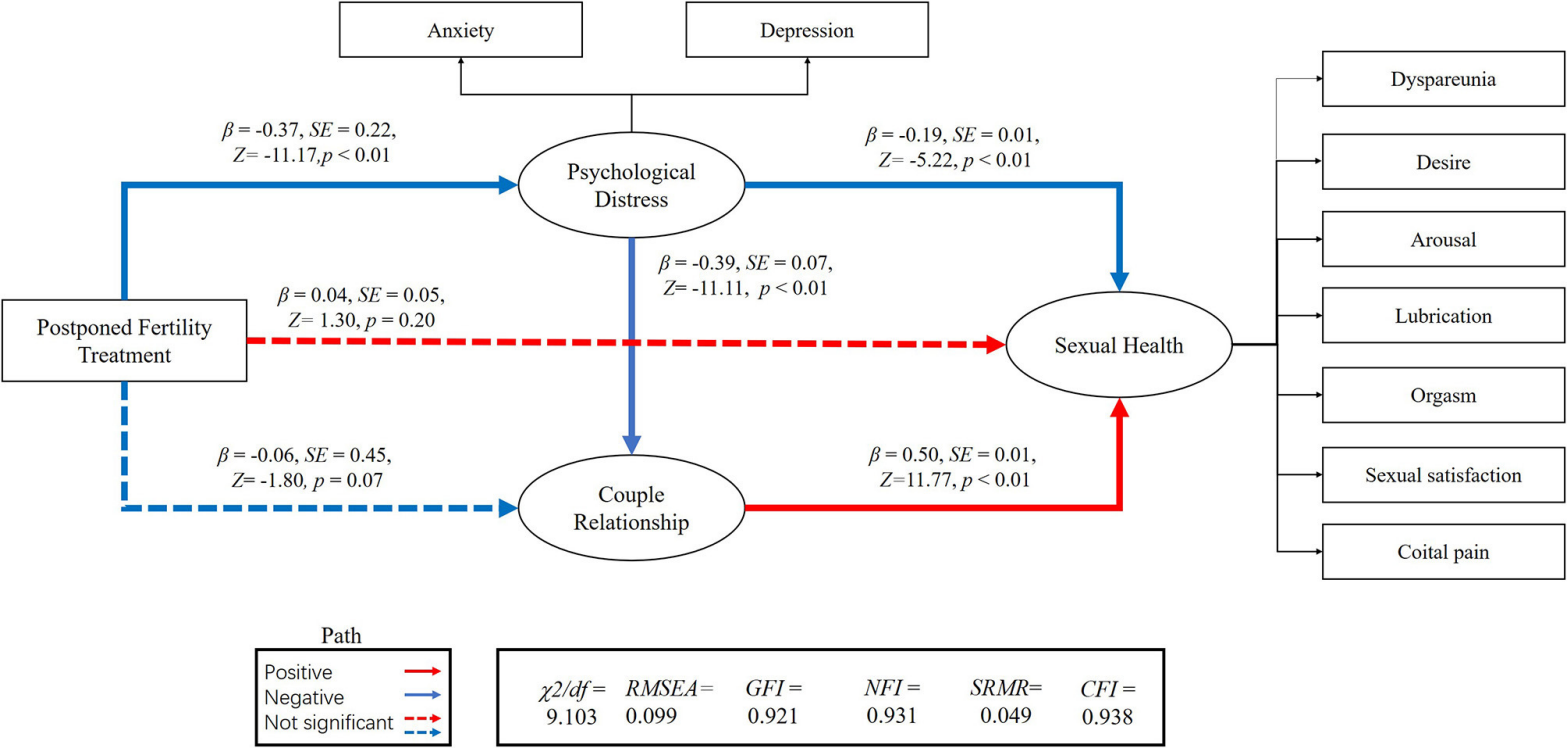
Association between postponed fertility treatment and sexual health mediated by psychological distress and couple relationship quality in women.

**Figure 2:**
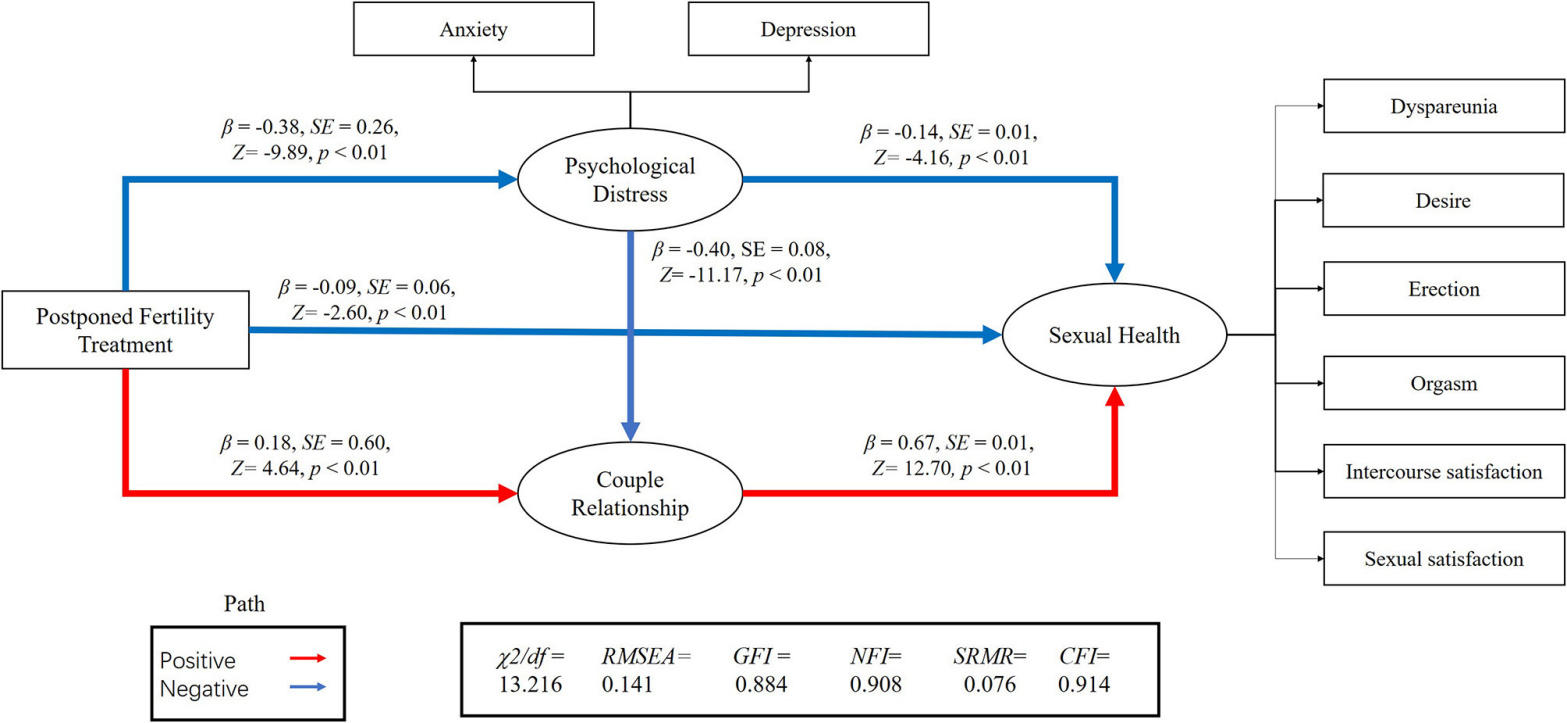
Association between postponed fertility treatment and sexual health mediated by psychological distress and couple relationship quality in men.

We performed an SEM to separately assess the relationship between postponed fertility treatments, psychological distress, couple relationship quality, and sexual health for both genders (Figures 1, 2). We used postponed fertility treatments (0 = increased, 1 = unchanged) as an exogenous variable, psychological distress (GAD-7 and PHQ-9 score) and couple relationship quality (QMI score) as mediator variables, and sexual health as the latent dependent (outcome) variables. Female sexual health was based on the six domains of the FSFI and incidence of dyspareunia (0 = almost always, 1 = usually, 2 = sometimes, 3 = rarely, 4 = very rarely, and 5 = never). Male sexual health was based on the five domains of the IIEF-15 and incidence of dyspareunia. Postponed fertility treatments did not significantly regulate sexual health in women (*β* = 0.04, *p* = 0.20), but had a negative regulate in men (*β* = −0.09, *p* < 0.01). Whereas, postponed fertility treatment significantly mediated levels of psychological distress in both genders (*β* = −0.38, *p* < 0.01 in men; *β* = −0.37, *p* < 0.01 in women), it only significantly mediated couple relationship quality in men (*β* = 0.18, *p* < 0.01) but not in women (*β* = −0.06, *p* = 0.07). Additionally, psychological distress had a direct negative effect on relationship quality (*β* = −0.40, *p* < 0.01 in men; *β* = −0.39, *p* < 0.01 in women) and sexual health (*β* = −0.14, *p* < 0.01 in men; *β* = −0.19, *p* < 0.01 in women), whereas relationship quality had a direct positive effect on sexual health (*β* = 0.67, *p* < 0.01 in men; *β* = 0.50, *p* < 0.01 in women; Figures 1, 2).

The association between postponed fertility treatment and sexual health was mediated by psychological distress and couple relationship quality in men (0.05 [95% CI: 0.04–0.14]; 0.12 [95% CI: 0.11–0.29]) and women (0.07 [95% CI: 0.06–0.15]; −0.03 [95%CI: −0.10–0.01]). Thus, the relationship between postponed fertility treatment and sexual health was partially mediated by psychological distress and relationship quality in men. However, only psychological distress fully mediated the association between postponed fertility treatment and sexual health in women. Interesting, both in men and women, postponed fertility treatment could affect sexual health *via* psychological distress and couple relationship quality (0.10 [95% CI: 0.12–0.22]; 0.07 [95% CI: 0.07–0.13]).

## Discussion

In this large-scale study, we found that postponed fertility treatment due to the COVID-19 pandemic had a significant impact on changes in sexual behaviour, psychological well-being, and couple relationships of patients with infertility. Through the SEM models, we observed that postponed fertility treatment could affect sexual health *via* psychological distress and couple relationship quality in both gender. Our study is the first to report the impact of postponed fertility treatment on the changes in sexual behaviour and sexual health of patients with infertility.

Although some researchers explored the impact of COVID-19 on changes in the sexuality of the general population (6, 7, 15, 20), drawing an overall consistent conclusion remains difficult. In a study conducted in Turkey, researchers found that a significant decrease in the sexual frequency and satisfaction in the pandemic period (34). Some people lost interest in sex completely, whereas some have exhibited an increase in sexual desire and used sexuality as a coping mechanism to relieve anxiety (35). Researchers believe studies from different countries and cultures are needed to clarify the effect of the pandemic and its consequences on sexual health (34). In our study, when the patients were grouped according to whether or not their fertility treatments were postponed, we observed that infertile patients who postponed treatment had significantly higher rates of change in sexual behaviour (sexual desire, sex frequency, sexual satisfaction, frequency of masturbation, and the use of pornography) than those who did not.

In this study, changes in frequency of masturbation and pornography use were obvious changes noted in the sexuality of infertile patients who delayed fertility treatment. Researchers believe that increased masturbation frequency is related to a decline in quality of life and sexual satisfaction (36), and frequent pornography use may also have a negative impact on sexual function and quality of life (37). A previous study reported that pornography was mainly used to relieve stress during the pandemic, to relieve loneliness and boredom (38), and to de-stress and release other pandemic-related negative emotions (39). Our study found that participants who postponed fertility treatment had more obvious symptoms of anxiety and depression than the control participants. Therefore, these changes in sexual behaviour observed in the patients who postponed fertility treatment may indicate how they cope with anxiety and depression.

A previous study has evaluated the moderating effects of sexual activity on mental health, relationship quality, and sexual health through SEM, and the relationship between them (14). Inspired by this, we explored the impact of delayed fertility treatment on sexual health through SEM. In our SEM, postponed fertility treatment has a direct effect on the occurrence of psychological distress, which is consistent with previous research stating that the interruption of fertility treatment causes anxiety in patients (10–13). Postponed fertility treatment could not directly mediate sexual function in women, but it could regulate it through two intermediary variables (psychological distress and relationship quality). Research also found that increased levels of anxiety and depression could lead to an unpleasant sex life or a sexual disorder, thereby inhibiting a person's interest in sex and further leading to sexual dysfunction. In turn, this could aggravate anxiety and depression in these individuals (40). Therefore, through the above SEM, we believe that postponed fertility treatment has an impact on sexual health.

Because our research was anonymous, we did not compare patients' hormone levels (which may have an impact on sexual function). To avoid this bias, we excluded patients with sexual dysfunction and those taking drugs that may affect their sexual function or mental state. Further studies about the impact of postponed fertility treatment on sexual health with consideration of different hormone values of infertile patients could be done. A new study found that COVID-19 infection and treatment had no long-term effect on male reproductive health, such as spermatogenesis and serum androgen levels (41).

These findings are meaningful for infertile patients, especially for patients who postponed fertility treatment in times of crisis. During infertility treatment, it is essential to highlight the sexual health and mental health of the patients. It is vital to detect these issues early in order to treat them appropriately with sexological and psychological therapy.

An important strength of this study is the large sample size; moreover, the participants were more willing to disclose private, sex-related information due to the anonymity of the questionnaire and privacy when completing the survey. Nevertheless, this study has its limitations. The main limitation is that we were unable to obtain quantitative data on the mental and sexual health of infertile patients prior to COVID-19. Thus, further large-scale longitudinal studies are needed to better understand the impact of postponed fertility treatment resulting from the pandemic on the mental and sexual health in patients with infertility.

## Conclusion

Our study found that postponed fertility treatment obviously affects patients' sexual and mental health and that it mediates sexual health by regulating psychological distress and relationship quality. We hope that this study will further emphasise the need for early screening and psychosocial intervention in the context of infertility to identify and prevent risk factors that may lead to the development of sexual dysfunction. Finally, it is important to detect these issues early on as they can be addressed with appropriate therapy in the form of sexological and psychological consultations, which could greatly benefit patients with infertility.

## Data Availability Statement

The original contributions presented in the study are included in the article/supplementary material, further inquiries can be directed to the corresponding author/s.

## Ethics Statement

This study was approved by the Shengjing Hospital Institutional Review Board for Research on Human Subjects (2020PS009F). Written informed consent for participation was not required for this study in accordance with the national legislation and the institutional requirements.

## Author Contributions

MD designed and executed the study, questionnaire evaluation, recruit patients, gathered, analysed, and interpreted the data, drafted the manuscript, and contributed to the critical discussion. YT guidance of questionnaire evaluation and statistical analysis. SW analysed and interpreted the data. FZ recruited patients, distributed questionnaires, and collected data. JT supervised, contributed to the study design, study execution, questionnaire evaluation, recruited patients, critical discussion, revised the manuscript, and approved the final submitted version. All authors have seen and approved the final version of this article.

## Funding

This study was supported by a grant from the National Key Research and Development Program (2018YFC1004203), the Major Special Construction Plan for Discipline Construction Project of China Medical University (3110118033), and the Shengjing Freelance Researcher Plan of Shengjing Hospital of China Medical University.

## Conflict of Interest

The authors declare that the research was conducted in the absence of any commercial or financial relationships that could be construed as a potential conflict of interest.

## Publisher's Note

All claims expressed in this article are solely those of the authors and do not necessarily represent those of their affiliated organizations, or those of the publisher, the editors and the reviewers. Any product that may be evaluated in this article, or claim that may be made by its manufacturer, is not guaranteed or endorsed by the publisher.
